# PReS-FINAL-2109: Genetic variations in patients with juvenile idiopathic arthritis and uveitis

**DOI:** 10.1186/1546-0096-11-S2-P121

**Published:** 2013-12-05

**Authors:** TH Reinards, H Albers, D Brinkman, S Kamphuis, M Van Rossum, E Hoppenreijs, H Girschick, C Wouters, R Saurenmann, R Toes, T Huizinga, J Houwing-Duistermaat, M Schilham, R Ten Cate

**Affiliations:** 1 Leiden University Medical Center, Leiden, The Netherlands; 2 Erasmus Medical Center, Rotterdam, The Netherlands; 3 Academic Medical Center, Amsterdam, The Netherlands; 4 Radboud University Medical Center, Nijmegen, The Netherlands; 5 University of Würzburg, Würzburg, Germany; 6 University Hospital Gasthuisberg, Leuven, Belgium; 7 University Children's Hospital, Zürich, Switzerland

## Introduction

Juvenile Idiopathic Arthritis (JIA) is accompanied by uveitis in approximately 20% of the cases. This is a serious complication with risk of impaired vision or even blindness. Both conditions are considered to be autoimmune diseases. Since uveitis is often asymptomatic, frequent ophthalmologic checks are needed to diagnose this complication at an early stage. Identification of risk factors for uveitis (besides presence of antinuclear antibodies (ANA)) could contribute to understanding of the pathogenesis of both diseases, and could be used as prognostic tool in an individual patient.

## Objectives

We investigated whether variations in candidate genes involved in autoimmunity are associated with the development of uveitis in JIA patients.

## Methods

Seventy European Caucasian patients with both JIA (all subtypes) and uveitis were included in this study. In 56 patients ANA were present, 5 patients were ANA negative, and in 9 patients ANA testing was inconclusive. Ninety-five single nucleotide polymorphisms (snps) on 52 loci were genotyped in cases and in 1598 healthy controls. Minor allele frequencies of these snps were compared between cases and controls.

## Results

Six of 95 snps were associated with JIA related uveitis (p < 0.05), 5 of which are on previously described susceptibility loci to JIA in general (*IL21*, *VTCN1*, 5q11, *PTPN22*, *AFF3*). A SNP in the promoter region of *IL1B *(rs16944), which was not associated to JIA in general in previous studies, was associated with susceptibility to JIA related uveitis (OR 1.63, 95%>CI 1.15 - 2.31, *p *= 0.006). None of the associations remained significant after Bonferroni correction for multiple testing.

## Conclusion

This study indicates a trend towards association of a genetic variant in *IL1B *with uveitis in JIA patients in particular. *IL1B*, coding for the proinflammatory cytokine IL1β, lies on chromosome 2q14, which harbours a cytokine gene cluster of nine related interleukin 1 family genes. Both genes and gene products of this cluster are associated with several autoimmune diseases, including experimental uveitis. IL1 inhibitors are used in the treatment of rheumatoid arthritis, (systemic) JIA, and sometimes uveitis. Although this result has to be replicated and finemapped in a larger and independent cohort, this study supports a role for the IL1 family in uveitis. Collection of long-term clinical follow-up data is ongoing, in order to compare JIA patients with uveitis to patients without uveitis to distinguish patients at risk from the others.

## Disclosure of interest

None declared.

